# Electronic pillbox-enabled self-administered therapy versus standard directly observed therapy for tuberculosis medication adherence and treatment outcomes in Ethiopia (SELFTB): protocol for a multicenter randomized controlled trial

**DOI:** 10.1186/s13063-020-04324-z

**Published:** 2020-05-05

**Authors:** Tsegahun Manyazewal, Yimtubezinash Woldeamanuel, David P. Holland, Abebaw Fekadu, Henry M. Blumberg, Vincent C. Marconi

**Affiliations:** 1 Addis Ababa University, College of Health Sciences, Center for Innovative Drug Development and Therapeutic Trials for Africa, P.O. Box 9086, Addis Ababa, Ethiopia; 2 Emory University School of Medicine and Rollins School of Public Health, Atlanta, GA 30322 USA

**Keywords:** Tuberculosis, Pillbox, Self-administered therapy, Directly observed therapy (DOT), Adherence, Treatment outcome, Trials, Sub-Saharan Africa, Ethiopia

## Abstract

**Background:**

To address the multifaceted challenges associated with tuberculosis (TB) in-person directly observed therapy (DOT), the World Health Organization recently recommended that countries maximize the use of digital adherence technologies. Sub-Saharan Africa needs to investigate the effectiveness of such technologies in local contexts and proactively contribute to global decisions around patient-centered TB care. This study aims to evaluate the effectiveness of pillbox-enabled self-administered therapy (SAT) compared to standard DOT on adherence to TB medication and treatment outcomes in Ethiopia. It also aims to assess the usability, acceptability, and cost-effectiveness of the intervention from the patient and provider perspectives.

**Methods:**

This is a multicenter, randomized, controlled, open-label, superiority, effectiveness-implementation hybrid, mixed-methods, two-arm trial. The study is designed to enroll 144 outpatients with new or previously treated, bacteriologically confirmed, drug-sensitive pulmonary TB who are eligible to start the standard 6-month first-line anti-TB regimen. Participants in the intervention arm (n = 72) will receive 15 days of HRZE—isoniazid, rifampicin, pyrazinamide, and ethambutol—fixed-dose combination therapy in the evriMED500 medication event reminder monitor device for self-administration. When returned, providers will count any remaining tablets in the device, download the pill-taking data, and refill based on preset criteria. Participants can consult the provider in cases of illness or adverse events outside of scheduled visits. Providers will handle participants in the control arm (n = 72) according to the standard in-person DOT. Both arms will be followed up throughout the 2-month intensive phase. The primary outcomes will be medication adherence and sputum conversion. Adherence to medication will be calculated as the proportion of patients who missed doses in the intervention (pill count) versus DOT (direct observation) arms, confirmed further by IsoScreen urine isoniazid test and a self-report of adherence on eight-item Morisky Medication Adherence Scale. Sputum conversion is defined as the proportion of patients with smear conversion following the intensive phase in intervention versus DOT arms, confirmed further by pre-post intensive phase BACTEC MGIT TB liquid culture. Pre-post treatment MGIT drug susceptibility testing will determine whether resistance to anti-TB drugs could have impacted culture conversion. Secondary outcomes will include other clinical outcomes (treatment not completed, death, or loss to follow-up), cost-effectiveness—individual and societal costs with quality-adjusted life years—and acceptability and usability of the intervention by patients and providers.

**Discussion:**

This study will be the first in Ethiopia, and of the first three in sub-Saharan Africa, to determine whether electronic pillbox-enabled SAT improves adherence to TB medication and treatment outcomes, all without affecting the inherent dignity and economic wellbeing of patients with TB.

**Trial registration:**

ClinicalTrials.gov, NCT04216420. Registered on 2 January 2020.

## Administrative information

**Table:** 

Title	Electronic pillbox-enabled self-administered therapy versus standard directly observed therapy for tuberculosis medication adherence and treatment outcomes in Ethiopia (SELFTB): protocol for a multicenter randomized controlled trial
Trial registration	ClinicalTrials.gov, ID: NCT04216420. Registered on 02 January 2020.
Protocol version:	04 December 2019, version 3
Funding:	The Fogarty International Center and National Institute of Allergy and Infectious Diseases of the U.S. National Institutes of Health under Award Number D43TW009127. The research content is solely the responsibility of the authors and does not necessarily represent the official views of the U.S. National Institutes of Health.
Author details:	Tsegahun Manyazewal^1^*, Yimtubezinash Woldeamanuel^1^, David P. Holland^2^, Abebaw Fekadu^1^, Henry M. Blumberg^2^, Vincent C. Marconi^2^ ^1^Addis Ababa University, College of Health Sciences, Center for Innovative Drug Development and Therapeutic Trials for Africa, Addis Ababa, Ethiopia ^2^Emory University School of Medicine and Rollins School of Public Health, Atlanta, Georgia, 30322, United States of America Study conception and Principal Investigator: TM. Initial study design and protocol: TM. Major contribution to study design and protocol: VCM, YW, DPH. Resource acquisition: HMB, AF. Draft the manuscript: TM. Reviewed and revised the manuscript: TM, VCM, YW, DPH, HMB, AF. Read and approved the final manuscript: TM, VCM, YW, DPH, HMB, AF.
Name and contact information for the trial sponsor:	Addis Ababa University - Email: health.sciences@aau.edu.et, Phone: +251118959055, website: www.aau.edu.et, P.O. Box: 9086, Addis Ababa, Ethiopia. The information and views set out in this study are those of the authors and do not necessarily reflect the official opinion of the Addis Ababa University

## Introduction

The World Health Organization (WHO) revealed its commitment to patients with tuberculosis (TB) in its End TB strategy that “everyone with TB should have access to the innovative tools and services they need for rapid diagnosis, treatment, and care; this is a matter of social justice, fundamental to our goal of universal health coverage.” This commitment is a collective responsibility towards human rights, making sure that no family is burdened with avoidable death or catastrophic expenses due to TB by 2030. However, taking pride in the slogan “End TB by 2030” is insufficient for true progress. In order to translate aspirational goals into reality, practical solutions should be sought. The strategy might appear to be a zero-sum if interventions on the disease neglect key socioeconomic burdens that individual patients are incapable of avoiding. The association between TB and poverty is a reality [1–5], but the disease is a global health security threat that urgently needs collective resources for mutual welfare [6, 7]. In the last three decades, various strategies and intervention packages have been formulated and implemented to halt the disease. However, improvements are not as expected [8, 9]; instead, a drug-resistant form of the disease is spreading [10–12], with globalization and migration fueling multiple strains worldwide [13–15]. TB remains the top cause of death worldwide from a single infectious disease and the major cause of death due to antimicrobial resistance. According to the 2019 WHO global TB report, an estimated 10.0 million people fell ill and 1.5 million people died from TB in 2018. Drug-resistant TB (DR-TB) continues to be a public health threat, where there were about half a million new cases of rifampicin-resistant TB, of which 78% had multidrug-resistant TB [16]. Africa accounts for one-quarter of new TB cases and TB-related deaths worldwide, with 2.5 million people falling ill and 417,000 people dying from TB annually. Of the total patients with TB co-infected with HIV globally, 72% of them live in Africa [17]. The true burden of DR-TB in the continent is poorly described, with only 51% of countries having formal data in the WHO global TB database, where DR-TB is largely missed and this requires a major effort to achieve the 2035 targets [16].

The main challenge is how to meet the End TB’s vision of “A world free of TB: zero deaths, disease and suffering due to TB” without bargaining the inherent dignity and economic wellbeing of patients with TB. It remains unclear how resource-limited countries would be able to meet one of the four key indicators of the strategy “Zero TB-affected families facing catastrophic costs due to TB by 2035” in situations where management of TB treatment still relies on directly observed therapy (DOT). Patients with TB from the poorer segments of society may not be benefiting from care delivery innovations: an apparent contradiction between “Global Commitment to End TB” and “reality on the ground.” Management of TB still largely depends on DOT. DOT has been viewed as an efficient strategy for adherence to treatment [18, 19], while evidence has demonstrated that it poses an economic and social burden to patients with TB and healthcare programs from low-income countries [20–25]. Treatment of TB requires at least 6 months, where patients in the intensive phase of DOT need to collect their medication at healthcare facilities daily and swallow tablets under the direct observation of a healthcare worker throughout the intensive phase [20, 21, 26].

To address the multifaceted problems associated with TB in-person DOT, the WHO recently recommended countries to maximize the use of digital adherence technologies (DATs). Such technologies, in general, have been able to improve TB treatment outcomes [27–30] and substantially reduce costs [31]. Yet, available data are limited for better conclusions of their effectiveness in various countries and settings [32–35]. In 2017, the WHO endorsed three DATs: short message service/mobile phone texting (SMS); Medication Event Reminder Monitor System (MERM); and video-supported directly observed therapy (VDOT) [36]. SMS involves sending a standardized and understandable text message to patients with TB regularly to remind and motivate them to take their prescribed medications. MERM is an electronic pillbox that records adherence to treatment, stores medication, emits audible and visual alerts to remind patients to take their medications, and enables healthcare providers to monitor adherence. MERM sleeves (99DOTS prototype) have an additional component that each medication blister is wrapped in 99DOTS envelopes to send to providers a hidden signal unrecognized by the patient. VDOT involves video communication between patients and healthcare providers, where providers watch patients take their medication, live or self-recorded, and provide advice and support. VDOT is mediated primarily through Internet-enabled smartphones, and Internet access is a critical component [36]. In general, there have been limited studies conducted on DATs. For existing studies, effectiveness within various low- and middle-income countries (LMIC) has been the focus of research and has resulted in some controversy. Table 1 summarizes a review of recent studies conducted on the three DATs.

**Table 1:** Summary of existing literature on TB digital adherence technologies

Reference	Country	Design	Outcome measure	Finding
*SMS*				
Bediang et al. 2018 [37]	Cameroon	RCT: SMS vs DOT	Treatment success, cure	No significant difference
Fang et al. 2017 [38]	China	RCT: SMS vs DOT	Treatment completion	High
Mohammed et al. 2016 [39]	Pakistan	RCT: SMS vs DOT	Treatment success, cure	No significant difference
Liu et al. 2015 [40]	China	RCT: 4 arms	Pill count, adherence	No significant improvement
Iribarren et al. 2013 [41]	Argentina	Cross-sectional	Feasibility and acceptability	acceptable and feasible
*MERM*				
Onwubiko et al. 2019 [42]	USA	RCT: MERM vs DOT	Treatment completion	Low
Park et al. 2019 [43]	Morocco	RCT: MERM vs DOT	Treatment success, cure	High
Liu et al. 2017 [44]	China	Multi-method	User performance, satisfaction	High
Broomhead et al. 2012 [45]	USA	Cross-sectional	Treatment outcome, cost	High, lower cost per patient
Thakkar et al. 2019 [46]	India	Cohort, 99DOTS used	Treatment adherence	High
*VDOT*				
Lam et al. 2018 [47]	USA	RCT. VDOT vs DOT	Treatment completion	High
Garfein et al. 2018 [48]	USA	RCT: VDOT vs DOT	Adherence, cost	High, lower cost
Nguyen et al. 2017 [49]	Vietnam	Prospective cohort	Treatment adherence	High
Chuck et al. 2016 [50]	USA	RCT: VDOT vs DOT	Treatment completion	High
Garfein et al. 2015 [51]	USA, Mexico	Single-arm trial	Treatment adherence	High in both settings

*DOT* directly observed therapy, *RCT* randomized control trial, *VDOT* video directly observed therapy

Considering the low-impact of SMS for adherence to TB treatment and the high investment and technology needed for VDOT, the advantage of MERM electronic pillboxes could surpass the other technologies, though more studies from LMIC are needed to inform its effectiveness. There are many electronic pillbox devices available in the global market; several were not endorsed by international health stakeholders (e.g. the WHO) or are not accessible in LMICs. Similar pillboxes have been in use for several decades for various medications. With the use of such an ordinary pillbox, patients can self-manage their medications, identify whether they have taken the dose, and minimize the rate of medication errors [52]. Previous studies found that individuals who used a pillbox had better adherence to treatment [53–55].

The evriMed500, manufactured by Wisepill Technologies, South Africa, is among the MERMs available on the market. The device is TB-appropriate technology allowing customization of the container for patients with drug-susceptible TB, MDR-TB, and TB-HIV [56]. It is currently being tested in several clinical trials and is being used clinically in India and China for patients with TB [57]. It costs less than US$10 per patient based on conservative reuse assumptions [58]. The evriMED500 dispenser consists of an electronic module and a medication container with three indicator lights/LEDs (green, yellow, and red). The green LED will flash once when the container is opened and again once when the container is closed, will quickly flash three times when the container is opened and closed quickly, will flash in sequence during the (daily) Medication Alarm, and will be on solid while connected via USB to a computer. The yellow LED indicated a need to refill medications and will flash with the green LED at the time of the Medication Alarm. If the Medication Alarm is not enabled, only the yellow LED will flash. The yellow LED will be on solid when the container is opened. The red LED flash indicates low battery power and will flash with the green LED at the time of the Medication Alarm. The red LED will be on solid when the container is opened. The device requires two AA batteries, which should last for more than 12 months. It can store more than 12,000 records/events.

In-person DOT is highly challenging for most patients with TB in sub-Saharan Africa. In these settings, DOT did not provide a significant solution for suboptimal treatment adherence. Instead, home-based and community-based therapies were shown to be possible alternative strategies to health-facility DOT according to studies conducted in Tanzania [59–61], Kenya [62, 63], Zambia [64], South Africa [65, 66], and Eritrea [67].

Ethiopia is among several countries highly encumbered by the TB epidemic and one of the least resourced in the world. According to the 2019 global TB report of the WHO, there were 113,613 TB cases reported in the country; and of the annual $94 million needed for TB care and control, the country’s domestic contribution was only 11% of this [17]. Despite TB care and treatment services being delivered free of charge, patients with TB face out-of-pocket payments [68, 69] and income losses [70] due to transportation, accommodation, and food to get treatment at a healthcare facility. This can be a major obstacle to adherence and has forced patients to stop working, sell their property, borrow money, and reduce their overall income [71]. These, in turn, have increased rates of loss to follow-up, disease relapse, and drug resistance [72]. Patients who travel daily to healthcare facilities for medication have also increased the transmission potential of the disease, especially in the capital city Addis Ababa, a highly congested city with overcrowded housing and public transportation [73]. Several studies conducted in Addis Ababa reported that patients with TB consider their daily DOT visits as worthless [74–78], and providers see DOT as a very challenging strategy for patients with TB [76]. As a result, daily DOT survives in principle, while implementation is irregular as both patients with TB and providers have uncertainties concerning the program. Providers report that patients with TB prefer taking the tablets at home once they have the necessary advice and counseling [74, 76]. Patients complain that they travel for daily DOT on foot under harsh road conditions for up to 2 h and 2.5 km, taking several rests on their way because of their sickness [74, 77, 79]. They spend substantial money on transportation [76, 80] and some lose their job due to work absences related to daily DOT [74, 78, 79]. Providers also report that patients appear exhausted and dissatisfied during DOT visits [74, 78]. Some patients claim that once they are initially informed of the disease, no-one talks with them on the subsequent DOT days; they just swallow the drug and return home [76]. Additionally, patients face stigma on their route to daily DOT and change their name in the TB clinic to disguise their identity [80]. The challenge is similar in other parts of Ethiopia [70, 81–83]. Such evidence discloses the difficulty of Ethiopia to meeting the overall End TB strategy targets by 2035 [84] unless significant investment is made to shape the current DOT strategy to a more advanced, technology-led, patient-centered strategy that could be responsive to individual patient preferences, needs, and values.

## Objectives

The main aim of this trial is to evaluate the effectiveness of pillbox-enabled SAT over standard DOT on adherence to TB medication and treatment outcomes in Ethiopia—a high TB burden country in sub-Saharan African. The secondary objectives are to assess the cost-effectiveness, thus individual and societal costs of treatment and quality-adjusted life years (QALY), when management of adherence followed pillbox-enabled SAT versus the standard DOT, and to assess the usability and acceptability by patients and their healthcare providers of the pillbox-enabled SAT.

## Methods

### Trial design

**Fig. 1:**
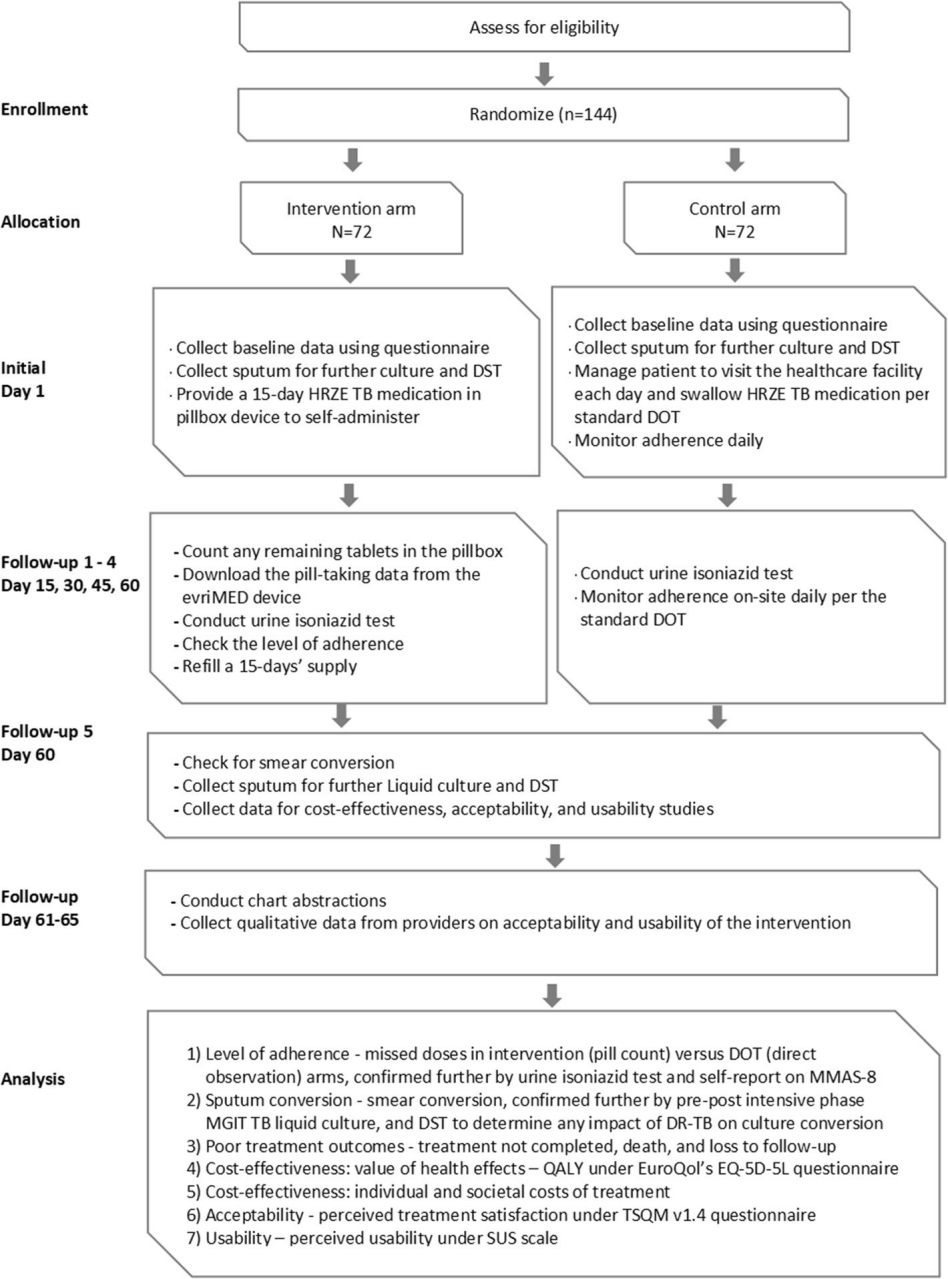
SPIRIT figure of the study

The study will be a prospective, multicenter, open-label, randomized, controlled, superiority, effectiveness-implementation hybrid, mixed-methods, two-arm trial. The study will not dictate diagnosis or treatment for TB; thus, it will not introduce or use new medications. The content of this protocol complies with the Standard Protocol Items: Recommendations for Interventional Trials (SPIRIT) guidelines [85] (see Supplementary file 1). Figure 1 is the SPIRIT figure summarizing the overall schedule and design of the trial.

### Study setting

Ethiopia is structurally divided into 11 autonomous administrative divisions. Addis Ababa, the capital, is among these administrative divisions and is the largest city of Ethiopia with the status of both a city and state. Addis Ababa is considered by some to be the capital of Africa, as it is a seat for the African Union headquarters and other international and regional organizations including the Africa Centers for Disease Control and Prevention. Administratively, Addis Ababa is divided into 10 sub-cities with distinct locations. As the city is crowded with population and housing, there is a high risk of TB transmission. The national health and health-related indicator [86] reports that in 2016, 2290 bacteriologically confirmed TB cases were identified in the city, of which 1811 (79.1%) completed their treatment and 1526 (66.6%) were cured. For these reasons, Addis Ababa was chosen as the study setting.

In Addis Ababa, a total of 94 public health centers provide TB care and treatment services under the DOT program. This study stratifies the 94 health centers on the bases of the 10 sub-cities where they are located. From each stratified group, one health center with the largest TB client load will be selected, with a total of 10 health centers to be included. Health management information system (HMIS) quarterly data (1 April–30 June 2019) taken from the Addis Ababa Regional Health Bureau will be used for the purpose. This will give a representative sample of study sites and participants. A TB clinic in each center will serve as the study site and primary location for patient contact for that center. Table 2 lists the 10 public health centers.

**Table 2:** Study sites

No.	Name of Health Center	Sub-city	No of. PTB cases bacteriologically confirmed or referred from other sites for initiation of TB treatment, 1 April–30 June 2019
	Addis Raey Health Center	Addisketema	41
	Akaki Health Center	Akaki kality	47
	Kebena Health Center	Arada	14
	Goro Health Center	Bole	31
	Adisu Gebeya Health Center	Gulele	22
	Kazanchis Health Center	Kirkos	19
	Alem Bank Health Center	Kolfe	38
	Teklehaymanot Health Center	Lideta	22
	Woreda 02 Health Center	Nifasilk lafto	53
	Woreda 13 Health Center	Yeka	35

### Eligibility criteria

The source population will be all new patients with TB symptoms who come to a study site and undergo bacteriological screening during the study period or TB patients bacteriologically confirmed elsewhere and referred to a study site for TB treatment.

Inclusion criteria will include:

- Patients with new, or previously treated, bacteriologically confirmed, drug-sensitive pulmonary TB
- Eligible to start the standard 6-month first-line anti-TB medication
- Outpatient status at the time of screening and enrollment
- Men or women aged 18–75 years, inclusive
- Able and willing to provide informed consent

The exclusion criteria will include:

- Patients with known DR-TB
- Any condition that causes cognitive impairment such as severe acute illness or injury, developmental retardation, or severe psychiatric illness and thus precludes informed consent or safely participating in the study procedures
- Inpatient status at the time of screening and enrollment
- Expected to move away from the study site or become incarcerated before the final study follow-up at month 2
- Concurrent extra-pulmonary TB
- Contraindicated medications
- Active liver disease that requires a TB regimen other than HREZ (isoniazid, rifampicin, pyrazinamide, and ethambutol)

The calculated total sample size is 144 participants with 72 in each arm. Considering a two-sided type-I error of 5%, probability of a type-II error of 20%, a power of 80%, 5% missing outcome data, and ±10% superiority from the current estimated adherence rate of 79% will provide 80% power to detect a difference between arms (90). The study will recruit and randomly allocate participants meeting the inclusion criteria to intervention and standard DOT arms. Because of the scope of the trial, the study staff and patients will not be blinded to group allocation.

In addition to patients with TB, the study will collect qualitative data from TB healthcare providers to assess further the usability and acceptability of the intervention. For this, the study will include healthcare providers who are currently providing TB treatment under DOT. The inclusion criteria are: a healthcare provider with academic qualification as a medical doctor, health officer (BSc), or nurse (BSc or Diploma); holds at least 3 months of experience providing DOT services in the facility; and able and willing to provide informed consent. With purposive sampling, the study will enroll 10 healthcare providers, selecting one provider from each of the 10 study sites.

### Interventions

Participants in the intervention arm will receive a 15-day TB medication supply (HRZE fixed-dose combination therapy of 15 doses) in an electronic pillbox device (evriMed500 digital medication monitoring and reminder device manufactured by Wisepill Technologies, South Africa) to self-administer. Providers will collect baseline data—including demographic, socioeconomic, behavioral, and social factors—using the study’s baseline questionnaire. Based on the baseline data, participants will be clustered into four behavioral determinants (use cigarettes, alcohol, Khat - a psychostimulant plant, and cocaine/marijuana) and three social determinants (homeless, unemployed, and illiterate) as appropriate for the purpose of analysis. Participants in this arm will return every 15 days, where the provider will count any remaining tablets in the pillbox, download the pill-taking data from the Wisepill device, evaluate the functionality of the device and troubleshoot as needed, and perform the urine isoniazid test. The level of adherence in their intensive phase of treatment will be calculated using the medication possession ratio (MPR) [87]. Any participant who misses more than five tablets in any 15-day refills will be reassigned to daily DOT throughout the remaining days of the intensive phase. Participants in the intervention arm can consult the healthcare provider in cases of medical illness or any adverse events outside of a scheduled visit before the next appointment. The phone number of the healthcare provider following their TB condition will be written at the backside of their appointment cards. The phone call strategy aims to maintain the DOT advantage for the intervention arm.

Participants in the control arm will get their treatment as per the standard practice of DOT, where participants will visit the healthcare facility each business day throughout the 2-month intensive phase to swallow their daily dose of HRZE with direct observation by the healthcare provider. Additionally, they will be given pills for the weekend to take them at home. The provider will collect baseline data, conduct a urine isoniazid test every 15 days, and cluster participants into behavioral and social determinants as applicable for participants in the intervention arm. For this arm, the management of participants who interrupt treatment will follow the national TB treatment guidelines.

Both arms will be followed up throughout the intensive phase which lasts for 2 months. The continuation phase (4 months) will follow the standard DOT practice for both arms. Both arms will have a TB care and treatment service free of charge, and the pillbox will be given to each participant in the intervention arm free of charge. For both arms, participants will receive treatment according to Ethiopian national TB treatment guidelines.

### Outcomes

#### Primary outcomes

The primary outcomes are adherence to medication and sputum conversion.

##### Level of adherence

For this study, the level of adherence will be defined as the proportion of patients who missed doses in intervention (pill count) versus DOT (direct observation) arms, confirmed further by urine isoniazid test (IsoScreen test, GFC Diagnostics Ltd., Bicester, England) [88] and a participant’s self-report of adherence on Morisky Medication Adherence Scale (MMAS-8, an eight-item structured, valid tool to measure self-reported adherence to medication) [89]. The time frame will be 2 months, with an assessment of missed doses every day, an IsoScreen urine test every 15 days, and a MMAS-8 assessment at the end of the 2 months. The WHO defines adherence to medication as the extent to which patients take their medications as prescribed with respect to dosage and intervals throughout the treatment period [90].

##### Sputum conversion

Sputum conversion will be defined as the proportion of patients with smear conversion following the intensive phase in the intervention versus DOT arms, confirmed further by pre-post intensive phase TB liquid culture (BD BACTEC MGIT 960 TB system, BD Diagnostics, MD, US) [91]. Pre-post treatment drug susceptibility testing will determine whether resistance to anti-TB drugs could have impacted culture conversion. The time frame is after the 2-month intensive phase. The updated (2016) Ethiopian National TB guidelines defines sputum conversion as smear conversion to negative after the end of the intensive phase [92].

#### Secondary outcomes

##### Adverse treatment outcomes

Adverse treatment outcomes will be defined as the proportion of participants in each arm having one of the following events: treatment not completed; death; or loss to follow-up.

##### Cost-effectiveness: value of health effects

QALYs will be calculated for each participant by arm using the EuroQol’s EQ-5D-5 L quality-of-life questionnaire [93], with discount rates of 3% and a score out of 100, with 100 defining optimal health. The time horizon will include the entire intensive phase. The questionnaire comprises five dimensions (mobility, self-care, usual activities, pain/discomfort, and anxiety/depression), with each dimension having five close-ended response levels. Average QALYs will be compared by study arm.

##### Cost-effectiveness: individual and societal costs of treatment

Individual and societal costs of treatment will be calculated for each participant using the Tool to Estimate Patients’ costs [94]. The Tool is a standard questionnaire developed jointly by KNCV Tuberculosis Foundation, WHO, and the Japan Anti-Tuberculosis Association. It comprises treatment costs (costs related to DOT and picking up TB drugs), guardian costs, and coping costs. The time horizon will include the entire intensive phase. Average costs will be compared by arm.

##### Acceptability

This is the proportion of participants in intervention versus DOT arms who perceived satisfaction with their TB treatment when measured on the Treatment Satisfaction Questionnaire for Medication scale (TSQM v 1.4) administered at the end of the intensive phase. The TSQM is a 14-item valid tool to assess participants’ satisfaction with medication across four domains: effectiveness; side effects; convenience; and global satisfaction [95]. The investigators will collect qualitative data from providers to supplement the acceptability study.

##### Usability

Usability will be calculated as the proportion of participants in the intervention arm who perceived the evriMED500 device as usable under adopted System Usability Scale (SUS, a 18-item valid tool to assess user performance of the device in six dimensions: ease of use; challenges; benefits; perceptions of being motivated; popularity; and recommendations) [96]. This assessment will occur at the end of the intensive phase. The investigators will collect qualitative data from providers to supplement the usability study.

### Data collection and management

The study investigators will identify healthcare providers in the TB clinics and provide training on the study procedures, how to operate the evriMED500 device, and how to perform a urine isoniazid test. The investigators will perform a 1:1 randomization of participants before the start of the study using computer-generated random numbers. Providers will enroll participants in the two arms sequentially as they arrive in the clinic and the investigators will routinely monitor the process. Providers will recruit participants and obtain written informed consent in the local language, which is Amharic. For both arms, the providers will collect baseline data, including demographic, socioeconomic, behavioral, and social determinants social using the study’s baseline questionnaire. For participants in the intervention arm, the providers will orient participants in the intervention arm as they enroll in how to use the evriMED500 device. The orientation time will depend on the efficiency of the participants to fully acquire and demonstrate the necessary skills. Providers will then dispense a 15-days TB medication supply (HRZE fixed-dose combination therapy of 15 doses) to participants within the evriMed500 device for self-administration. Providers will collect sputum specimens from participants, write their cell phone numbers at the backside of participants’ appointment cards to communicate in cases of medical illness or any adverse events before the next appointment, and inform them to return every 15 days. When participants returned, the providers will count any remaining tablets in the pillbox, download the pill-taking data from the evriMED device, check functionally of the device, and conduct a urine isoniazid test. The providers will fill out the study’s adherence follow-up form to capture information if the drugs are taken every day and, if not, the reasons for non-adherence. The providers will then evaluate the level of adherence based on the preset criteria, and refill a 15-day TB medication supply in the same pillbox as appropriate. The providers will collect sputum specimens from participants at the end of the intensive phase.

For the control arm, providers will handle participants according to the standard DOT procedure, where participants will visit the healthcare facility each business day in the intensive phase to swallow their daily dose of HRZE with direct observation by the provider. The providers will fill out a similar adherence follow-up form for the control arm. Additionally, if a participant in the control arm requests medications for self-administration, the provider will collect information about the date requested and for how many days requested.

At the end of the intensive phase, trained research experts will complete several data instruments for all participants. The first is a self-report of medication adherence that the experts will administer using the eight-item MMAS-8 questionnaire. The second is a case report form for which data will be extracted from TB registration logs and participants’ charts, focusing on overall treatment outcomes and side effects. For the third instrument, the experts will administer the EuroQol’s EQ-5D-5 L quality-of-life questionnaire to all participants. The experts will also assess costs using the Tool to Estimate Patients’ Costs. Then the experts will administer the TSQM version 1.4 assessment. Finally, the experts will administer the 18-item SUS tool to determine user performance for intervention participants. Following the collection of data from participants, the study investigators will collect qualitative and quantitative data from the providers to learn more about the usability and acceptability of the intervention from providers and the healthcare system perspectives.

For the laboratory research involving biological specimens (urine and sputum), the study will use WHO-approved diagnostic tools. The IsoScreen test is a semi-quantitative urine test that provides a reliable and immediate indication of adherence to isoniazid-containing treatment regimens for patients with TB. It uses the reagents of the Arkansas Method—barbituric acid (20 mg), potassium cyanide (10 mg), and chloramine-T (10 mg)—in an enclosed plastic testing device for safe and rapid testing in clinics and patients’ homes. The providers will perform this test within the study facilities for both arms every 15 days, thus four times per participant. Sputum specimens collected before and after the intensive phase will have MGIT TB liquid culture and DST tested at Armauer Hansen Research Institute (AHRI). Initiation, termination, or completion of treatment will rely only on the standard procedures available at the study sites, which could be acid-fast bacilli or Xpert MTB/RIF assay. Thus, the outcomes of the study’s laboratory results will not dictate the standard diagnostic or treatment procedures.

The study will use a password-protected offline Research electronic data capture (REDCap) database (Vanderbilt University, Nashville, TN, USA) [97] to enter data and store entered data in an encrypted drive. The Principal Investigator will keep the source data in a locked cabinet at the study’s central office. The assigned study staff will check a random sample of 10% of all data entry forms for entry errors.

### Statistical analysis

The study team will develop a formal statistical analysis plan according to the intention-to-treat principle; the team will develop this plan during the course of the study but prior to a review of any major analyses. The analysis will principally include t-tests, Fisher’s exact two-tailed tests, and mixed-effect regression models to determine differences between the intervention and DOT arms. All of the quantitative statistical tests will consider a *p* value of < 0.05 to indicate significant associations. For each standardized tool used, their standard scoring algorithm will be normalized for specific demographic characteristics and used for calculations. The qualitative data obtained from individual interviews with healthcare providers will be analyzed using content analysis based on a theoretical framework.

### Ethical considerations

The study obtained formal ethical approval from the Institutional Review Board of the Addis Ababa University College of Health Sciences (ID: 077/19/CDT). The protocol is registered with ClinicalTrials.gov (NCT04216420) and the WHO’s International Clinical Trial Registry Platform (ICTRP). Study investigators completed human research participation training and good clinical practice training. All study sites will provide the necessary permissions and written concurrence for data collection. Each participant will provide written informed consent before enrollment. An independent Data and Safety Monitoring Board will oversee the study. Any study staff responsible for the conduct, management, or oversight of the trial will complete good clinical practice training. Any amendment to the protocol will be reviewed and approved by the IRB before the changes are implemented to the study. In addition, all changes to the informed consent form will be IRB-approved; a determination will be made regarding whether a new consent needs to be obtained from participants who provided consent using a previously approved informed consent form.

The study does not involve a new investigational product; therefore, no exemption will be sought. The pillbox device has been in use for clinical purposes other than TB. Treatment of TB will not deviate from the national guidelines, neither the type of regimen nor the dose, but only the management of treatment.

Participants will not receive compensation, either in cash or in kind, as this would influence the outcomes of the study. However, if the participants are coming just for the purpose of the study, they will be reimbursed for transport.

### Oversite and monitoring

#### Interim monitoring and analysis

The investigators will conduct interim monitoring and submit an analysis report to the independent Data and Safety Monitoring Board (DSMB). Then, the report will be sent together with DSMB’s recommendation to the IRBs. The DSMB will periodically review and evaluate the study’s collected data to follow up participant safety, the accuracy of study procedures, and the study progress in order to provide recommendations on the continuation, modification, or termination of the study. The DSMB will consist of an expert in the clinical aspects of TB, an expert biostatistician, and an investigator with expertise in current clinical trials conduct and methodology. External monitors may conduct technical audits, before, during, and after completion of the trial. This will include reviewing the research protocol, operations manual, standard operating procedures, and training materials before initiation of the trial. The site investigators will make study documents and pertinent records readily available for inspection by the local IRB and site monitors.

#### Participant discontinuation

There will be premature study discontinuation if there is refusal of study participants to participate in all components of the study; a request by the participants to withdraw, request from the healthcare providers if s/he thinks the study is no longer in the best interest of the participants; or at the discretion of the IRB/Ethics Committee, regulatory bodies, sponsor, or consensus of the investigators.

#### Unexpected or adverse events

Given that the study is neither dictating the need for treatment of TB or specific regimen, serious adverse events will not be directly attributable to the study. The study will not dictate diagnosis or treatment algorithms for TB, and all diagnoses testing assays and treatment regimens will follow the Ethiopian national guidelines; thus, it will not introduce or prescribe new drugs. The major risks of this screening program are related only to pill-taking mechanisms. Adverse reactions to TB medications will not be considered the outcomes of the study. The investigators will capture these events only to the extent they are available in the study health facilities’ records and registries. The investigators will conduct chart abstractions to review adverse events that are related to TB treatment, as recorded from the TB clinic registries. For patients who are co-infected with HIV, the chart review will also include the type of HIV regimens, viral load, CD4 counts, and other co-infections diagnosed and treated.

Regarding TB diagnosis, at the initial stage or after the intensive phase, there could be discrepancies between the health facility results (Smear microscopy or Xpert MTB/RIF) and the study result (MGIT liquid culture). In this case, the investigators will communicate the results to the healthcare providers for their review and decision.

## Discussion

This study will be the first in Ethiopia, and of the three in sub-Saharan Africa, to evaluate the effectiveness of pillbox-enabled SAT as a multicenter randomized controlled trial. The study will provide evidence of whether pillbox-enabled SAT is superior to the standard DOT for medication adherence and treatment outcomes. The study will assess whether digital adherence intervention is cost-effective, usable, and acceptable. The study targets patients with TB living in Ethiopia, a high TB burden LMIC in sub-Saharan Africa where alternative TB treatment strategies to in-person DOT do not exist.

If effective, this approach could substantially improve adherence to treatment, increase sputum conversion, reduce patient-side costs due to daily DOT, and improve quality of life. It could also have a strong public health impact by reducing transmission of the disease to healthcare providers and the community at large as the approach can reduce patients’ daily travels to healthcare facilities for in-person DOT. This approach will provide patients with TB the freedom and ownership of their treatment, which ultimately reduces mortality and morbidity thereby contributing significantly to the End TB Strategy. The findings will enable patients, healthcare providers, and policymakers to make informed decisions about the value of the intervention.

The study intends to use several clinical, biomedical, behavioral, and economic measurement tools to learn extensively about the desired outcomes of interest. The assessment of adherence using electronic, self-report, and biological specimens will provide credible and reliable justification of results. The diagnostic tools we plan to use are WHO-approved, and the questionnaires are valid and psychometrically sound for use in a local context. The study will collect original data from both patients and their TB care providers in local facilities, providing substantial evidence on the usability and acceptability of the intervention in real-world settings.

## Trial status

- Protocol date and version: 4 December 2019, version 3, approved by the Institutional Review Board of the College of Health Sciences, Addis Ababa University on 13 November 2019
- Recruitment start: 1 March 2020
- Recruitment end: 1 February 2021

## Supplementary information

**Additional file 1.** SPIRIT 2013 Checklist.

**Additional file 2.** Participant information sheet and consent form (English version).

## Data Availability

Not applicable.

## Abbreviations

DAT: Digital adherence technologies
DR-TB: Drug-resistant tuberculosis
DST: Drug susceptibility testing
DOT: Directly observed therapy
HRZE: Isoniazid, rifampicin, pyrazinamide, and ethambutol
MERM: Medication Event Reminder Monitor System
MOST: Multiphase Optimization Strategy
QALY: Quality-adjusted life year
SAT: Self-administered therapy
TB: Tuberculosis
VDOT: Video-supported directly observed therapy
WHO: World Health Organization
