# Supplementary material for: Electronic pillbox-enabled self-administered therapy versus standard directly observed therapy for tuberculosis medication adherence and treatment outcomes in Ethiopia (SELFTB): protocol for a multicenter randomized controlled trial
Source: Trials. 2020 May 5;21:383. doi: 10.1186/s13063-020-04324-z (PMC7201596; doi:10.1186/s13063-020-04324-z)
Supplement: Supplementary file 2 — Additional file 2. Participant information sheet and consent form (English version). [file 13063_2020_4324_MOESM2_ESM.docx]

**Participant information sheet and consent form (English version)**

**Information sheet**

Study title: Electronic pillbox-enabled self-administered therapy versus standard directly observed therapy for tuberculosis medication adherence and treatment outcomes in Ethiopia: a multicenter randomized controlled trial (SELFTB trial)

**What is this study about?**

This is a research project being conducted by Addis Ababa University and Emory University. We are inviting you to participate in this research project because you are a patient prescribed anti-TB drugs in this facility, you will be on these drugs for at least two months, and this study is to describe poor adherence and factors that influence adherence among persons on anti-TB treatment. This study purpose is to evaluate the use of pillbox-enabled self-administered therapy compared to the standard DOT strategy for improving medication adherence and treatment monitoring.

**What will I be asked to do if I agree to participate?**

If you agree to participate in the study, you will be asked questions by an interviewer who will ask you questions while and recording your responses into the questionnaire. The study will be conducted in this health facility, and it will take an average of about 40 minutes for the interviewer guided questionnaire to be completed.

If you agree to be in the study, you may have from one to three interventions. We will kindly ask you some questions about your personal and social life, which may take about 15 minutes. For your TB treatment, you will be randomized to be assigned in either of the two arms. In this study, we use an electronic pillbox devise to assist patients’ medication management. Patients will take a 15 days dose and return to the TB clinic every 15 days for re-fills and for giving urine specimen. However, you can come out of schedule visits or phone call and consult the healthcare provider in cases of medical illness or any adverse events before the next appointment. The phone number of the healthcare provider who will be following your TB condition will be written at the backside of your appointment card. The box we provided is only used for containing TB drugs. Please keep the box from water, children and fire. Electronic medication box is free for patients who are enrolled in the study. Some TB patients in this facility are receiving the standard DOT methods, so that we will compare treatment adherence amongst patients who take their medicines at home and patients receiving the drugs in the standard DOT method. We will give you full instruction of how you use the device. In your second visit after 30 days, we will ask you some questions about your experience using the device, and if it helps to minimize patients’ and health care facilities’ costs. Using urine test, we will confirm further if you adhered with your medication.

**Would my participation in this study be kept confidential?**

We will do our best to keep your personal information confidential. To help protect your confidentiality, we shall assign a code instead of your name, your name will not appear on any data collection instrument, the data collected will be kept in a confidential place under lock and key and will require a secret code in order to be accessed on the computer. Through the use of an identification key, the researcher will be able to link your survey to your identity; and only the researcher will have access to the identification key. If we write a report or article about this research project, your identity will not be revealed.

**What are the risks of this research?**

There may be some risks from participating in this research study. These include staying longer as you are being interviewed, or getting your service a little late because of your participation in the study after having the questionnaire completed.

**What are the benefits of this research?**

This research is not designed to help you personally, but the results may help the investigator learn more about determinants of anti-TB treatment adherence. There will be no payment for participation, either in cash or in kind, to patients in the intervention or DOT arm, or healthcare providers, as this would influence outcomes of the study. However, if the participants are coming just for the purpose of the study, they will be reimbursed for transport. We hope that in the future, other people will benefit from this study through better understanding and improved policies of anti- TB treatment especially as regards to adherence to treatment, and the best way to implement these policies to achieve best results for people on anti-TB treatment.

**Do I have to be in this research and may I stop participating at any time?**

Your participation in this research is completely voluntary. You may choose not to take part at all. If you decide to participate in this research, you may stop participating at any time. If you decide not to participate in this study or if you stop participating at any time, you will not lose any benefits to which you otherwise qualify.

**Is any assistance available if I am negatively affected by participating in this study?**

Any assistance that you may need if negatively affected by the research study procedures will be accorded you such as counselling and referral for care according to the standard procedure.

**What if I have questions?**

This research is being conducted by Dr Tsegahun Manyazewal at Addis Ababa University. If you have any questions about the research study itself, please contact Dr Tsegahun at: P. O. Box 9086, Addis Ababa, Ethiopia. Tel +251 11 878 7311, and E-mail [tsegahunm@gmail.com.](mailto:tsegahunm@gmail.com.) Should you have any questions regarding this study and your rights as a research participant or if you wish to report any problems you have experienced related to the study, please contact:

**Institutional Review Board of the College of Health Sciences, Addis Ababa University:**

Tel: +251 11 896 1396, P.O. Box 9086, Addis Ababa, Ethiopia

**Institutional Review Board, Addis Ababa Health Bureau**

Tel: P.O. Box 180908, Addis Ababa, Ethiopia

**Consent form**

Study title: Electronic pillbox-enabled self-administered therapy versus standard directly observed therapy for tuberculosis medication adherence and treatment outcomes in Ethiopia: a multicenter randomized controlled trial (Self-TB trial)

Principal Investigator: Dr Tsegahun Manyazewal

Co-Investigators: Prof. Vincent C. Marconi, Dr Yimtubezinash Woldeamanuel, Dr David P Holland

Date in Ethiopian calendar (DD-MM-YY):☐☐-☐☐-☐☐

Study Clinician ID: ☐☐☐☐ Participant ID: ☐☐☐☐☐☐

Participant Full Name: ____________________________________________________________

The research staff of the Addis Ababa University, Center for Innovative Drug Development and Therapeutic Trials for Africa (CDT-Africa), and Emory University School of Medicine have planned to perform the Self-Tuberculosis (TB) study to see if electronic pillbox-enabled self-administered therapy (SAT) is more useful that Directly Observed Therapy (DOT) for TB medication adherence and treatment outcomes.

I have read/has been read to me that the purpose of the study is to evaluate the use of pillbox-enabled self-administered therapy compared to the standard DOT strategy for improving medication adherence and treatment monitoring. The study team will use information from the study to learn how to improve medication adherence and treatment outcomes in Ethiopia.

I have understood that if I agree to participate in the study, I may be in the control or intervention phase.

I have been assured that all my clinical and personal information taken from me will be kept confidential. I have also been informed that participation in the study could end if I refuse all study procedures or if the Institutional review board or the study team terminates the study.

Participation in the study is voluntary and I can withdraw my consent at any time without any change to my medical care and treatment I need at the health facility as usual. If I have questions about the study, I can contact the head of this health facility, the investigators of the study (Tel: +251 11 878 7311), or the Institutional review board (Tel: +251 11 896 1396).

I have been allowed to ask questions and they have been answered. I have received a copy of this consent document to keep.

**I agree to participate in the study: ☐**Yes **☐**No

Signatures…………………………………………..Date………………………………

**Study participant**

Signature……………………………………………Date………………………………

**Witness**

Signature……………………………………………Date………………………………

**Study Clinician**
